# Blinded by the Light: Artificial Light Lowers Mate Attraction Success in Female Glow-Worms (*Lampyris noctiluca* L.)

**DOI:** 10.3390/insects12080734

**Published:** 2021-08-17

**Authors:** Mira Van den Broeck, Raphaël De Cock, Stefan Van Dongen, Erik Matthysen

**Affiliations:** Evolutionary Ecology Group, Campus Drie Eiken, University of Antwerp, Universiteitsplein 1, Wilrijk, B-2610 Antwerp, Belgium; rdecock@hotmail.com (R.D.C.); stefan.vandongen@uantwerpen.be (S.V.D.); erik.matthysen@uantwerpen.be (E.M.)

**Keywords:** *Lampyris noctiluca*, glow-worms, light pollution, mate attraction, female mating success

## Abstract

**Simple Summary:**

Nocturnal light pollution is a worldwide growing problem, threatening nocturnal biodiversity. We studied the impact of streetlights on mating success of the female common glow-worm, a bioluminescent nocturnal beetle (*Lampyris noctiluca* L.) that uses light signals for sexual communication. We monitored individual females daily and assumed that when they stopped glowing, they had effectively mated. We found that females in dark surroundings typically stopped glowing after one night, while females in illuminated areas glowed for significantly more nights, in some cases up to 15 nights. Our study confirms previous hypotheses that females exposed to artificial light suffer from a reduced mate attraction success, which can lead to population declines. Our findings represent valuable information that can be used by policy makers and managers to conserve the iconic glow-worms.

**Abstract:**

Nocturnal light pollution from anthropogenic origin is increasing worldwide and is recognised as a major threat for nocturnal biodiversity. We studied the impact of artificial light on the mate attraction success of female common glow-worms (*Lampyris noctiluca* L.) by daily monitoring their glowing status in the field, acting as a proxy for mating status throughout the mating season. We found that females in dark surroundings typically stopped glowing after one night, indicating that they had mated, while females in illuminated areas glowed for significantly more nights, in some cases up to 15 nights. Our study confirms previous findings and hypotheses that females exposed to artificial light suffer from a reduced mate attraction success with a negative impact on populations.

## 1. Introduction

Artificial light at night (ALAN) is a globally occurring threat to wildlife, which has emerged relatively recently with the expansion of human industrialisation [1,2,3,4] and is currently still increasing worldwide by 6% every year [5]. ALAN mostly affects nocturnal species [4,6,7], however, diurnal species and even plants can also be affected [8]. Eventually ALAN may affect entire ecosystems [1,2]. Even in protected areas, which can successfully act as a buffer to expanding urbanisation and deforestation, the effects of light pollution are tangible [9].

A particular group of nocturnal animals that are especially vulnerable to artificial light are the bioluminescent glow-worms and fireflies (Lampyridae), due to their light based signals for mating [2]. A recent global survey indicated ALAN as the second most important threat to glow-worm and firefly populations worldwide, after habitat loss and fragmentation [10], with significant negative impacts on reproductive success and population trends [1,2]. Several studies on firefly species with flashing displays have found negative effects on courtship behaviour (among others: [11,12,13,14,15]). Studies on the effects of ALAN on adult common glow-worms (*Lampyris noctiluca* L.) are scanter compared to their flashing North American and Asian counterparts. In *L. noctiluca* only the flightless females emit a continuous bioluminescent signal for the flying males. Ineichen and Rüttimann [16], Bird and Parker [17], Stewart et al. [18], Elgert et al. [19], and Van den Broeck et al. [20] studied the effects of light intensity on mate seeking behaviour of males. They found that different kinds of artificial lights induced a lower capture rate of males in female-mimicking traps [16,17,18,19,20] even at strikingly low light intensities [20]. Dreisig [21] also found that incandescent and fluorescent lamps reduced the circadian glowing activity in females. Ineichen and Rüttimann [16] furthermore observed high numbers of females displaying in illuminated areas. Because females cease to glow once mated [22], it was suggested that high numbers of glowing females actually indicated lack of mating success [16]. Moreover, given the short mating period it is likely that many of these females would die without having mated. Gardiner and Didham [23] found the opposite pattern with increasing numbers of glowing females away from ALAN but were not able to discriminate between actual female abundance and glowing activity. More recently, Elgert, Hopkins, Kaitala, and Candolin [19] studied female response to artificial light in a laboratory set-up and found that females did not move away from the light, but were less likely to glow and hid away more. However, no studies have documented the actual mating success of females in relation to ALAN.

In this study we monitored individual females daily in artificially lit and unlit surroundings, covering an entire mating season, to test whether streetlighting (low pressure sodium (LPS); monochromatic orange) reduces female mate-attraction success. We thereby used the cessation of glowing as a proxy for female mating [22,24]. We also introduced field-collected and captive-raised females to different light environments in order to confirm that any effects on mating success were directly caused by ALAN, and not due to differences in female quality or attractiveness between areas with more or less light.

## 2. Materials and Methods

### 2.1. Study Area

This study was carried out in Lippelobos (51°02′09.7″ N 4°14′53.0″ E), and Speelbos Marselaere (51°02′27.4″ N 4°16′18.5″ E), which are situated in Lippelo, Belgium. Both are open mixed deciduous forests mostly dominated by beech (*Fagus* sp.) and chestnut (*Castanea* sp.) trees with little undergrowth. Both are bordered by roads equipped with LPS streetlighting as well as unlit roads. Candidate areas were visited in spring 2019 to verify the presence of glowing larvae as a predictor of high glow-worm abundance. The fieldwork was carried out from 24 June until 13 July 2019, which covered the entire flight season of males [20]. The beginning of the reproductive season was monitored by setting up light-lure traps (as described in Van den Broeck, De Cock, Van Dongen and Matthysen [20]) each night to determine when the first males were flying.

### 2.2. Light Environments

**Figure 1:**
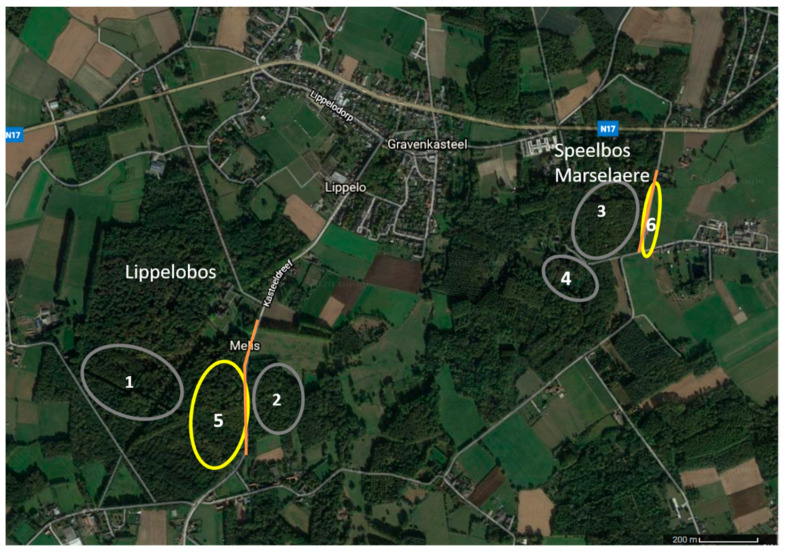
Map of the locations used. The dark locations are indicated in grey (nr 1–4) and the illuminated locations in yellow (nr 5 and 6). The orange lines represent the lit streets.

Adult female glow-worms were monitored both in dark areas and areas illuminated to a varying degree by LPS streetlights. A female was considered to be located in a lit area when the artificial light was visible to the human eye. The measured light intensities in the lit areas (at the location of glowing females) varied between 0.017 and 8.53 lux. One illuminated area in Lippelobos (nr 5 on Figure 1) consisted of an open beech stand bordering a lit road with little undergrowth and a thick leaf litter layer. The second illuminated area in Speelbos Marselaere (nr 6 on Figure 1) consisted of a grassy roadside (about 2 m wide), along both sides of a paved road lit by LPS streetlights with woodland on one side and horse pastures on the other side. The dark environments (0.008–0.020 lux) were clearly distinguishable from the illuminated areas as no visible artificial light reached those areas. The dark locations were more diverse and consisted of locations with more dense undergrowth (nr 1, 2, and 4 on Figure 1) or open forest as described for Lippelobos (nr 3 on Figure 1). Some dark areas were located relatively close to illuminated streetlights (see Figure 1), however due to dense vegetation and orientation of the lamps these areas were clearly dark. In total, 3 females were monitored in area 1, 11 females in area 2, 1 female in area 3, 9 females in area 4, 21 females in area 5, and 6 females were monitored in area 6.

### 2.3. Study Species

The common glow-worm is widespread in Flanders and is found in open forests and forest edges. During the day, female larvae search for vegetation bordering open spaces where they will later pupate [25]. This location will then remain their displaying site until they have mated [16]. During this courtship display, females may climb on the extremities of ground vegetation and expose their lantern. They emit a continuous lime-green glow and start their display just after sunset, when the ambient light is low (about 1.0 lux), for two to three hours until a male finds her, and mating occurs. After mating, the female lays eggs and dies [21,24]. If a female does not succeed to attract a mate, she will glow again the next nights, remaining at the same location until she is found by a male, or dies [26]. During the day, adult females hide in the forest litter or under the vegetation [21,27]. We can thus assume that the glowing status can be used as a proxy of mating status.

A total of 51 females were monitored, whereby we gradually increased the number of monitored females every night, as the initial handling to mark and measure females took some time. Twenty-eight adult females were found in situ, of which 19 were monitored in the location where they were initially found, and 9 were relocated to a different ambient light condition, mostly moving them from lit conditions to dark (*n* = 8) and in one case between lit conditions, either within or among different locations. Twenty-three females were raised in captivity from field-collected larvae and released in different light conditions (11 in lit areas and 12 in dark areas). These females were collected as larvae in April 2019 on humid and warm nights (above 10 °C) and pupated in captivity to be used later for the experiment. For the females that were not raised in captivity, the day on which they started to glow was unknown because of the great number of simultaneously glowing females each night rendering it impossible to identify every female of the study area, and because other experiments were done on the same nights limiting the time for monitoring the entire population.

### 2.4. Monitoring

**Figure A1:**
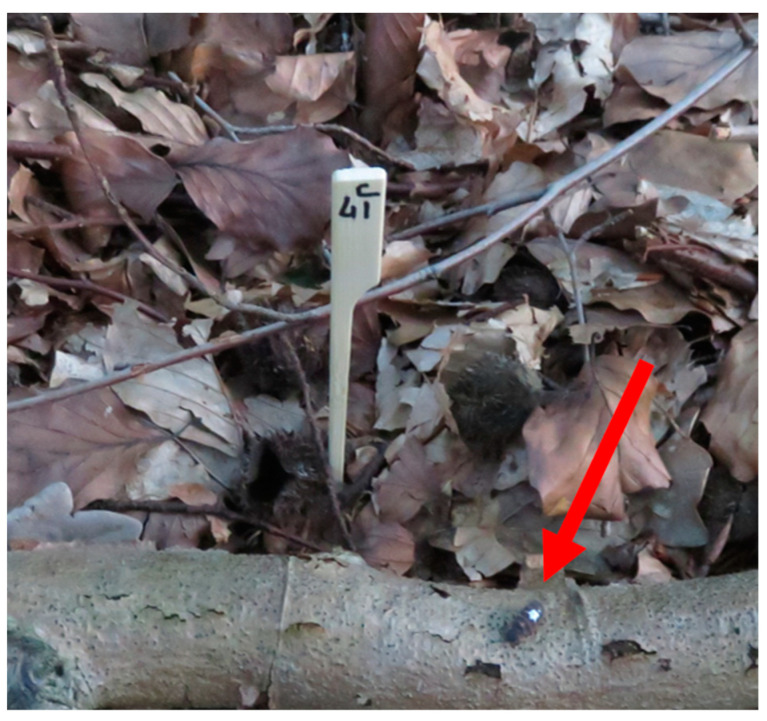
Marked female in the field next to a wooden stick with individual marking.

The glowing status of individual females was checked every night between 26 June and 13 July 2019, between 10.00 P.M.–12:00 A.M., which corresponds to their natural glowing period [28]. The in situ found females were arbitrarily selected for monitoring as we entered the study areas. The captivity-raised females were also released at arbitrarily chosen places. The monitored females were weighed and marked with a paint pen (DecoColor™) with a white dot on their back at first discovery in the field or when released for females raised in captivity. The light intensity was measured near each female with a luxmeter (Skye^®^ SKL 310), with the sensor oriented to the nearest streetlight if there was one. A small wooden stick was placed next to the female in order to find them back again the following nights, as illustrated in Figure A1. Adult female glow-worms are indeed extremely sedentary and rarely move from their chosen display site [19,24]. If a female was not found on her location, this was considered as a glowing cessation.

### 2.5. Statistical Analysis

The statistical analysis was performed using R [29]. A survival analysis was performed using the *survival* package [30] and *survminer* package [31]. The time elapsed until a female stopped glowing was taken as an analogy of the time to mortality used in survival analysis. A female was considered to have stopped glowing when she was not seen aglow for a minimum of two consecutive days, to account for rare occasions where a female resumed glowing after one day. The survival analysis allowed us to take into account censored data of females that were still glowing by the end of the monitoring period, and also allowed to include females that entered the experiment at different times. The logarithm of ambient light intensity (in lux), the date, date squared, female origin, and its interaction with light intensity and female weight were used as explanatory variables. Female weight was added in the model because Hopkins et al. [32] found that males preferred the brightest dummy females and that female glowing brightness correlates with a higher female weight and a higher fecundity (but see Borshagovski et al. [33]). The date and date squared were added as a factor after being converted in Julian date to take into account the nonlinear change in male densities over the mating season. We also included the origin of females (found in situ, translocated, raised in captivity) and its interaction with light intensity, to verify that results were not biased by a possible association between female attractiveness and light environment. A stepwise model selection was then performed.

## 3. Results

**Figure 2:**
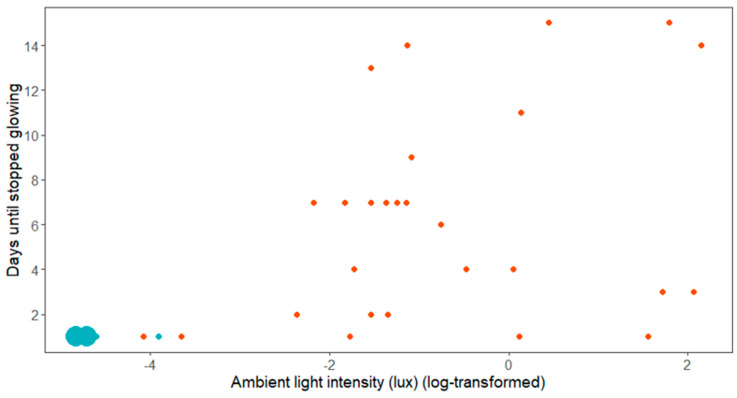
Observed time until mating (using cessation of glowing as a proxy) in relation to light conditions. Blue dots are the females in dark areas and red dots are the females in illuminated areas. The size of the dots reflects the number of individuals. Note that not all females were monitored until they ceased glowing. The two large dots in the lower left corner represent 22 females (N_tot_ = 51).

The females which were located in dark areas all stopped glowing after one day (*n* = 24, including 4 females found in situ, 12 females raised in captivity, and 8 females translocated from lit areas), whereas the females in illuminated areas glowed for a longer period with a median of 6 consecutive nights (range 1 to 15, *n* = 27, 15 females found in situ, 11 females raised in captivity and 1 female translocated from a lit area). Anecdotally, two females located in an illuminated area were even found glowing 20 and 24 days after their first discovery in the field, after the regular monitoring had ended. As Figure 2 shows, all females located in light environments below approximately 0.1 lux (corresponding to a log value of −2.36) stopped glowing after a single day.

The survival analysis confirmed that time to mating increased significantly with ambient light intensity (*p* > 0.001; estimate = −0.598 + S.E = 0.125). Julian date squared (*p* = 0.76), Julian date (*p =* 0.19), the interaction of female origin with light intensity (*p* = 0.39), female weight (*p =* 0.10) and origin (*p* = 0.11) had no significant effect and were consequently removed from the model. The final model only contained light intensity as significant factor.

## 4. Discussion

Daily monitoring of female glow-worms revealed that females exposed to LPS streetlighting glowed for significantly more nights than females located in natural darkness. Females in dark environments ceased to signal after a single night which we assumed to indicate that they had mated. The fact that eight females relocated from illuminated to dark areas also all stopped glowing after one night supports the idea that this is due to the direct effect of artificial light rather than an association between the light environment and female quality or attractiveness. More generally, the survival analysis confirmed that female origin had no impact on the relation between light environment and time to mating. The short glowing period of females in dark environments is in line with a study on a non-light-polluted glow-worm population where females glowed for a mean period of 1.7 to 3.4 nights, and half of the females glowed for just one night [24]. On several occasions we found one or more males attempting to copulate with a female in dark surroundings less than two hours after she was released, which we never observed with females in the illuminated areas. Since males appear somewhat later and disappear earlier in the season than the females [34], mating success of females may vary throughout the mating season [24]. Captures of males in the same area show that they were present throughout the period of female monitoring, although numbers of males were clearly low in the first and last days of the study (Figure S2 in Van den Broeck, De Cock, Van Dongen, and Matthysen [20]). However, we found no effect of date on time to mating in this study, possibly because numbers of males and unmated females varied in a similar way throughout the study period; however, we did not make standardised counts of glowing females to corroborate this.

Females located in illuminated areas glowed for many more nights, which can be explained by several non-mutually exclusive hypotheses including (1) repulsion (males are repulsed by light of high intensity), (2) light adaptation (bright lights decreases male visual sensitivity), or (3) the wash-out effect (female signals are invisible to males due to a decreased contrast with their surroundings) [2,35]. First, males could be repulsed from very bright light intensities close to the light source. Repulsion from high light levels (from 200 lux on) has been reported once under multiple light colours in male *L. noctiluca* [36]. However, it is also possible that at low light intensities males are actually attracted to the orange light emitted by the LPS streetlights. Indeed, Bek [37] (unpublished thesis) found that males aggregated on roads beneath orange LPS streetlights, and an unpublished pilot experiment [38] suggested that orange LED light attracted males at close distance. Nonetheless, even if males are present in dim orange light, they are still not able to locate females. Secondly, the light adaptation hypothesis suggests that the photoreceptors of the highly sensitive compound eyes of male glow-worms might adapt to overexposure to unnaturally bright artificial lights, which reduces the sensitivity to the small lights of females [18]. Finally, the wash-out effect implies that strong artificial light reduces the contrast between the female’s signal and her surroundings, making her appear at least less bright or even invisible to males [4]. If the wash-out effect is responsible for our findings, this could imply that males cannot discriminate between orange/red and green light colours. This has already been confirmed in other Lampyridae [39]. Indeed, numerous other Lampyridae have been shown to have only two photoreceptors: one sensitive to short wavelengths (blue and UV light) and one with a sensitivity tuned to the female signalling emission spectrum [40,41,42,43]. Behavioural evidence suggests that this is also the case for *L. noctiluca* as the addition of a red light in a green light stimulus had no impact on the attractiveness of the stimulus [44]. The light adaptation hypothesis could be tested on males by presenting a female-simulating green LED illuminated from above by increasingly bright green light, while the wash-out effect can be tested by simultaneously presenting a green and red LED light and comparing the attraction to both colours.

An extended glowing period has several costs for females. First of all, it has an energy cost as glow-worms are capital breeders and cease to feed once adulthood is reached and then rely on the resources built up during their larval stage [26,33,45]. An extended glowing period has indeed a cost on fecundity as the quantity of eggs decreases with time [26,46]. It has even been hypothesised that females can use their unlaid eggs as a direct energy source [47]. Additionally, females that glow for a longer time are likely to have a higher predation risk as they are more exposed to potential predators such as toads, frogs, bats, spiders and birds [32,45,48,49]. Nevertheless, predation rates may be relatively low since *L. noctiluca*, like many lampyrids, are distasteful to numerous predators due to a substance which is structurally similar to lucibufagins [50,51]. More importantly, given the short flight season, there is a considerable risk that females exposed to ALAN may die without having mated at all. This may be problematic on a population level if a large part of the glow-worm population is affected. Ineichen and Rüttimann [16] suggested this may eventually lead to population sinks where populations can no longer sustain themselves and only persist through immigration from higher-quality habitats [52].

Our data show that mate attraction may already be reduced by light intensities at a threshold intensity between 0.02 lux and 0.1 lux (Figure 2). Below this intensity, all females stopped glowing after one night. In the same study area but using an experimental setup, we were able to identify a comparable threshold intensity of 0.027 lux beyond which white LED light has negative effects on the mate finding ability of the males [20]. As a comparison, typical ALAN intensities at street level range between 10 and 60 lux [53], and we measured street level light intensities of 20.8 lux and 11.6 lux beneath two street lamps in our study area. According to our measurements, spill light levels of around 0.1 lux may be recorded up to 100 m away from the streetlights. Incidentally, this corresponds to the recommendation made by Gardiner and Didham [23] to create buffers between glow-worm populations and light sources. This recommendation was based on counts of glowing females, although in their study it is not clear to what extent these counts reflect actual female abundances.

By daily checking the females, we noticed that some females stopped glowing for a night and then resumed glowing. We saw this only in the areas exposed to artificial light. It is known that under natural conditions, females usually glow continuously from dusk when the ambient illumination is low (about 1.0 lux or lower), for two to three hours until they have mated [21], and typically without interruption over the course of several nights [24]. However, experiments by Dreisig [21] using fluorescent lights and incandescent lamps, showed that light intensities of 2.5 lux induced a shorter glowing duration, while higher illuminations of 10 lux completely inhibited female glowing activity. Elgert, Hopkins, Kaitala, and Candolin [19] similarly found that females delayed or refrained to glow under artificial light of 1.5 lux and that artificial light increased the probability that the females would hide away. This could be an explanation for the fact that some of our females located in illuminated areas showed a disrupted glowing pattern. Since females were only checked once each night, we were unable to say if the females only temporarily ceased to glow during the night, or if they remained the whole night without glowing. In either case could the disrupted glowing pattern further reduce mating probability.

It appears intriguing that females do not move out of apparently unsuitable illuminated habitats, given that they have some ability to move. Last instar female glow-worm larvae seek an appropriate location to pupate during the daytime whereby they search for open spaces such as forest clearings, roads and railway lines crossing the forests [16,54], and move away from forest edges for a maximum of 200–300 m [55]. Once pupated, adult females do not seem able to further assess the suitability of their location in terms of ambient light conditions, and rarely move further than 10–30 cm from their initial location [16,22,27,55,56]. We also never observed females more than one meter away from the first location. Elgert, Hopkins, Kaitala, and Candolin [19] confirmed in laboratory conditions that females do not move away from a light source and considered this maladaptive behaviour, since only a small displacement could considerably increase their mating success. Our study provides an in situ validation of this statement as we showed that females translocated to dark environments had an improved mate attraction success.

In the context of the conservation of glow-worm populations, it remains crucial to further clarify the role of light intensity and emission spectra on glow-worm behaviour and breeding. LED technology allows to produce monochromatic light of any desired spectral composition [57], which could be used to produce outdoor illumination which is the least harmful to nocturnal wildlife. Currently, long wavelength (which corresponds to red/orange light) outdoor lighting are being recommended worldwide as ecologically sustainable alternatives to white light because they are considered least harmful to numerous nocturnal animals [1,58]. This makes the finding that monochromatic orange light emitted by LPS streetlights is strongly disruptive for mate attraction and mating success of female glow-worms, even at low light intensities, very alarming. Indeed, there is not one spectral composition which is unharmful for every nocturnal species [1]. Even in the Lampyridae, there does not seem to be a single neutral spectral composition for different species, and for *L. noctiluca* there seems to be no harmless light colour at all. Red lights seemed the least harmful for *Aquatica ficta* fireflies courtship behaviour [59]. However, in an unpublished pilot experiment, we found that *L. noctiluca* males were attracted to orange light and away from a green LED simulating a female [38], also disrupting the female attraction success. Furthermore, Booth, Stewart, and Osorio [44] have shown reduced male attraction towards a green light stimulus when combined with blue light in *L. noctiluca*. Blue light furthermore had an inhibitory effect on male activity in an unpublished pilot experiment [38]. Blue lights were also found to affect negatively the courtship signalling behaviour of *A. ficta* fireflies [59]. Broad spectrum white lights have also been shown to negatively impact the mate finding success in *L. noctiluca* [17,18,19,20]. Thus, it might yet become difficult to find types of outdoor lighting that will suit human visibility in brightness and spectral composition whilst still being unharmful to nocturnal organisms in general [60]. In this regard it would be highly valuable to identify intensity thresholds and light emission spectra that attenuate the negative impacts of artificial lights, to suggest clear recommendations for policy makers. Nevertheless, the most efficient solution would be to turn off, reduce or shield outdoor lighting near ecologically vulnerable areas, in particular for the comparatively short duration of the mating period.

## Data Availability

The data presented in this study are openly available in Figshare at doi:10.6084/m9.figshare.15060273.
